# Coping in Chronic Tinnitus Patients

**DOI:** 10.3389/fneur.2020.570989

**Published:** 2020-11-19

**Authors:** Sebastiaan M. Meijers, Arno F. Lieftink, Inge Stegeman, Adriana L. Smit

**Affiliations:** ^1^Department of Otorhinolaryngology, Head and Neck Surgery, University Medical Center Utrecht, Utrecht, Netherlands; ^2^Brain Center Rudolf Magnus, University Medical Center Utrecht, Utrecht, Netherlands

**Keywords:** tinnitus, coping, THI, TQ, CISS

## Abstract

**Background:** Tinnitus is the perception of sound without an external stimulus. A large part of the adult population experiences this symptom but never seeks professional help, where others have devastating complaints in daily life. This suggests that the impact of tinnitus varies among patients and may be influenced by coping strategies and multiple psychological factors.

**Method:** Cross- sectional study of patients visiting the tertiary tinnitus referral center of the University Medical Center Utrecht, the Netherlands. Three hundred and twenty-one consecutive chronic tinnitus patients were evaluated by the tinnitus care group Utrecht from 6–2007 till 11–2012 of which 308 patients were included. Patients completed two tinnitus distress questionnaires (THI, TQ), a coping questionnaire (CISS) as well as a psychopathological questionnaire (SCL-90-R).

**Results:** Emotional-orientated coping and distraction-orientated coping strategies were significantly correlated with the experienced tinnitus burden. Also a significant negative correlation with task orientated coping was found. The effect size was small. Tinnitus distress also had a significant positive correlation with anxiety, agoraphobia, depression, insufficiency of handling, distrust & personal sensitivity, hostility and sleeping problems.

**Conclusion:** Patients with higher tinnitus handicap scores demonstrated the use different coping strategies than the patients with lower distress scores. This insight in coping strategies in a group of patients with high tinnitus burden is useful for counseling patients. As tinnitus coping strategy might be related to the extent and success of habituation, this outcome could be of interest to optimize tinnitus treatments in the near future.

## Introduction

Tinnitus is the perception of sound or noise without the existence of an external acoustic stimulus (1, 2). It is estimated that ~30% of individuals perceive tinnitus at some point in their life (2). Tinnitus is a complex condition, in which many components are responsible for perceived burden such as tinnitus related sleep and concentration problems. The large differences in the tinnitus itself (etiology, lateralization, temporal course, and sound characteristics) results in a heterogonous condition whereby the burden that patients experience is divers and the individual needs of patients for tinnitus related health care are various (3, 4).

In tinnitus patients, negative affective states are reported (5–7). Negative affective states like depression and anxiety are associated with a lower quality of life (QoL) (8). Besides this, the subjective distress tinnitus causes is demonstrated to be lower in patients who have higher tinnitus acceptance (9, 10). Therefore, differences in the experienced distress and QoL in people with tinnitus could be explained by the way patients cope with their tinnitus.

Coping is referred to as an individual's effort to regulate stressful situations (11). It is suggested that coping styles have a major influence on the way individuals react to these situations and negative life events (12). Lazarus and Folkman (11) developed a model which recognizes two types of coping: problem focused coping responses and emotion-focused coping responses. Subsequently, Endler and Parker (13) added a third coping style; avoidance coping.

So far, several researchers tried to investigate the relationship between experienced tinnitus distress and different coping strategies. Correlations were found between “maladaptive” coping (14–17) and a passive coping style and the experienced tinnitus handicap (18). Though, from these studies strong conclusions are hindered by unclear definitions of coping and the lack of usage of validated coping questionnaires (5). Further insight in the relation between coping strategies and the experienced effect of tinnitus on daily life could provide opportunities for improvement of current therapeutic strategies.

Therefore, we aim to investigate the correlation between the experienced tinnitus burden and the used coping strategies in a cohort of chronic tinnitus patients.

## Materials and Methods

### Study Population

This study is a prospective cross-sectional cohort study performed by the Tinnitus Care Group of the department of otorhinolaryngology of the University Medical Center Utrecht (UMCU), The Netherlands. Patients with complaints of tinnitus of more than 2 months attended the care group from June 2007 till November 2012, and were studied following a structured diagnostic protocol. The patients were referred to the department by general practitioners and otolaryngologists from local hospitals for diagnosis, counseling and treatment of their tinnitus (3).

As part of their evaluation by the Tinnitus Care Group, the patients completed questionnaires regarding personal and tinnitus characteristics, experienced tinnitus handicap, coping strategies and psychological symptoms before their psychological intake. Patients who did not return the questionnaire booklet were excluded from this study. All patients gave informed consent and all patient data collected through this protocol was anonymized and entered in a database. This study was performed in accordance with the Declaration of Helsinki. Exemption for full review from the Local Research Ethics Committee of the UMC Utrecht, The Netherlands, was obtained for this study (local number 12-611/C).

### Baseline Characteristics

Baseline characteristics were gathered including age, gender, level of education categorized in high level (university, college education, Higher General Secondary Education), middle level (post-secondary college) and low level (lower General Secondary Education or primary school). Tinnitus characteristics were collected using Visual Analog Scales (VAS) with a score 0–10 for perceived burden (0 = no burden, 10 = extreme burden). Tinnitus loudness (0 = inaudible, 10 = extreme loud), Tinnitus pitch (0 = extreme low, 10 = extreme high) and the time present (0 = constantly absent, 10 = constantly present) as well as tinnitus related difficulties experienced in concentration (0 = no problem concentrations, 10 = extreme concentration problems), sleeping (0 = no sleeping problems, 10 = extreme sleeping problems), irritation (0 = no irritation, 10 = extreme irritation), social life (0 = no problems in social life, 10 = extreme problems in social life), family life (0 = no problems in family life, 10 = extreme problems in family life), work/study (0 = no problems at work, 10 = extreme problems at work) were measured using a 0–10 Likert scale. Also the number and location of perceived sounds were scored. Hearing level was assessed by pure-tone audiometry performed by trained audiology assistants. The testing was done in a soundproof cabin with Telephonics TDH 39 earphones and according to ISO 389 standards on a Decos audiology audiometer (Decos Technology Group, Noordwijk, The Netherlands). Mean hearing levels were calculated as the averaged combined mean hearing loss of both ears in dB of 0.5–1 to 2–4 kHz.

### Tinnitus Burden Questionnaires

To measure the individual experienced tinnitus burden the Tinnitus Handicap inventory (THI) (19) was used. THI is a self-report questionnaire consisting of 25 questions focusing on tinnitus related distress. Every question can be answered with no (zero points), sometimes (two points) and yes (four points). The total score ranges between 0 and 100 and can be divided in five categories (0–16 points: slight tinnitus, 18–36 points: mild tinnitus, 38–56 points: moderate tinnitus, 58–76 severe tinnitus and 78-100 catastrophic tinnitus). Subscales of the THI encompass a functional subscale (eleven items), the emotional subscale (nine items), and the catastrophic subscale (five items). The Dutch version of the questionnaire was used.

The Tinnitus Questionnaire (TQ) (20) is a self-reported questionnaire focusing on physical, emotional, and the social effects caused by tinnitus. It contains 52 questions with three answer possibilities: true (zero points), partly true (one point), and not true (two points). The questions are divided in six subscales (emotional and cognitive distress, intrusiveness, auditory perceptual difficulties, sleep disturbance, and somatic complaints). Combining the answers of the questions results in a score with a range from 0 to 84, with higher scores indicating more distressing tinnitus.

### Coping Questionnaire

The Coping Inventory for Stressful Situation (CISS) (13) is a self-response questionnaire consisting of 48 questions to measure three basic coping strategies with 16 items per scale; task-orientated coping, emotional-orientated coping and avoidance-orientated coping. The patient rates questions on a 5-point scale from 1 to 5, depending on applicability (1 = not at all, 5 = very much). Task-orientated coping, emotion-orientated coping and avoidance orientated coping were all investigated using 16 questions (minimum total score 16, maximum total score 80). Avoidance orientated coping can be divided in two components: distraction and social diversion. Distraction was measured using eight questions (minimum score 8, maximum score 40). Social diversion was measured using five questions (minimum total score five, maximum total score 25). The remaining three items of the avoidance scale involve both or neither social diversion or distraction. When using task-orientated coping, stressful situations are treated as a problem that needs to be solved, were emotional orientated coping is aimed at mitigating emotional stress. Avoidance-orientated coping style encompasses postponement of dealing with the current problem (21). Scores for all items per scale are summed to form scale scores; higher scores indicate a greater use of that particular coping strategy. Different norm scores for males and females exist and are based on a study in adult females and males out of the general population, with reference scores as described in the questionnaire manual (13, 22).

### Psychopathology Questionnaires

The Symptom CheckList-90 Revised (SCL-90R) (23) is a 90-item self-response questionnaire used to screen for general psychiatric symptomatology. The test can be used for both dimensional evaluation and to screen for psychopathological aspects in non-psychiatric patients. Participants rate the degree to which they have experienced each of the symptoms during the past week on a five-point rating scale. For this study the Dutch version of the SCL-90R was used (24), measuring the following dimensions: anxiety, agoraphobia, depression, somatization, insufficiency of handling and thoughts, distrust and interpersonal sensitivity, hostility and sleep problems. The total and sub scores out of the presented study will be compared to a norm score of the general population (24).

### Statistical Analysis

For statistical analysis IBM SPSS statistics version 25.0 was used. The results were compared to norm scores (13, 22). To analyze the correlation between tinnitus burden and psychological outcomes measures by the SCL-90R and CISS, linear regression analysis was performed. A value of *p* < 0.05 was defined as statistically significant. Missing data was imputed using SPSS statistics version 25.0 using a total of 10 imputed datasets. This study is reported according to the Strengthening the reporting of observational studies in epidemiology statement (STROBE) (25).

## Results

### Patient Characteristics and Tinnitus Distress

From June 2007 till November 2012 a total of 321 patients visited the Tinnitus Care Group Utrecht. 308 patients returned their questionnaire booklets and were included in this study. The group consisted of 208 (67.5%) males and 100 (32.5%) females (Table 1: baseline characteristics). The mean age was 51.8 years (range: 17–84 years). Most of the patients had a high level of education (51.3%). The mean VAS score of tinnitus burden was 6.8 on a Likert scale ranging from 0 to 10. The mean THI score was 45.6 (SD 22.85). Missing data was present in several questionnaires; THI: 5/308, CISS: 23/308, SCL-90R: 22/308. The mean hearing loss was 24.84 dB (SD 23.61) (0.5–1 to 2–4 kHz). The reported tinnitus location was right side 13%, left side 17%, twice unilateral (when a different sound is heard in each ear) 13%, bilateral 25%, “in the head” 24%, and varying locations 8%.

**Table 1:** Baseline characteristics.

	**Number of patients total *n* = 308 (%)**	**Mean/SD/[min-max]**	
Age (years)	308 (100)	51.78/12.35/[17–84]	
Gender	308 (100)		
Male	208 (67.5)		
Female	100 (32.4)		
Level of education	199 (100)		
High	102 (51.3)		
Middle	44 (22.1)		
Low	53 (26.6)		
**Tinnitus characteristics**			**Interpretation of score:**
VAS burden:	226 (73.4)	6.76/2.09/[0–10]	0 = no burden, 10 = extreme burden
Number of sounds:	233 (75.6)	1.91/1.07/[0–6]	
Loudness	272 (88.3)	5.98/2.37/[0–10]	0 = inaudible, 10 = extreme loud
Pitch ^*^	262 (85.1)	6.34/2.40/[0–10]	0 = extreme low, 10 = extreme high
Time present ^*^	264 (85.7)	8.31/2.40/[0–10]	0 = constantly absent, 10 = constantly present
Concentration	263 (85.4)	4.44/2.82/[0–10]	0 = no problem concentrations, 10 = extreme concentration problems
Sleeping	264 (85.7)	4.31/3.21[0–10]	0 = no sleeping problems, 10 = extreme sleeping problems
Irritation	273 (88.6)	4.37/2.89/[0–10]	0 = no irritation, 10 = extreme irritation
Social	265 (86.0)	4.18/3.05/[0–10]	0 = no problems in social life, 10 = extreme problems in social life
Family life	256 (83.1)	3.25/2.85/[0–10]	0 = no problems in family life, 10 = extreme problems in family life
Work/study	206 (66.9)	4.51/3.02/[0–10]	0 = no problems at work, 10 = extreme problems at work
Hearing level (dB)^**^	293 (95.1)	24.84/23.61/[−5–155]	
THI	303 (98.4)	45.64/22.85/[2–98]	
TQ	301 (97.7)	39.63/16.69/[3–77]	

*Tinnitus Characteristics are scores using 0–10 Likert scales*.

* *noise number one*.

** *Mean pure tone average hearing level (0, 5, 1, 2, 4 kHz) of both ears*.

### Scores of Psychological Questionnaires

Outcome of the SCL-90 and CISS questionnaires are summarized in Table 2.1. For both men (M) and women (F) the mean CISS scores of the tinnitus patients demonstrated average-low scores compared to norm scores in the categories task orientated coping [M 55.35 (SD 10.80), F 55.00 (SD 11.30)], avoidance orientated coping [M 39.54 (SD 9.59), F 43.57 (SD 9.89)], and social diversion [M 13.81 (SD 3.64), F 15.67 (SD 4.28)]. Men also had an average-low mean score in emotional- orientated coping [33.38 (SD 9.86)], were women scored a mean average [37.96 (SD 10.10)]. The use of distraction as coping strategy scored a mean average in comparison to the norm [M 18.16 (SD 5.22), F 20.54 (SD 6.20)]. The SCL-90R questionnaire, which was used to screen for psychiatric symptomology, displayed higher than norm mean scores for anxiety [15.69 (SD 6.25)], depression [26.43 (SD 9.82)], somatization [20.74 (SD 7.59)], insufficiency of handling and thoughts [15.20 (SD 6.10)], sleeping problems [7.43 (SD 3.51)] and overall psychoneurotic score [137.91 (SD 39.93)].

**Table 2.1:** Results.

**Psychological questionnaires:**	**Sex**	**Mean**	**SD**	**Range**	**Mean norm score (SD) (22)**
**CISS (*****N*** **=** **290, 194 M, 96 F)**					
Task oriented [16–80]	M	55.35	10.80	16–78	59.80 (8.39)
	F	55.00	11.30	16–78	60.92 (8.94)
Emotion– oriented [16–80]	M	33.38	9.86	16–78	36.70 (10.14)
	F	37.96	10.10	16–61	40.21 (10.67)
Avoidance–oriented [16–80]	M	39.54	9.59	16–57	46.62 (9.76)
	F	43.57	9.89	19–74	48.23 (9.68)
Distraction [8–40]	M	18.16	5.22	8–30	19.13 (5.88)
	F	20.54	6.20	8–45	21.91 (5.75)
Social Diversion [5–25]	M	13.81	3.64	5–23	15.18 (4.00)
	F	15.67	4.28	6–24	17.34 (4.25)
**SCL−90–R (*****N*** **=** **299)**					**Norm score** **(****24****)**
Anxiety [10–50]		15.69	6.25	10–45	12–14
Agoraphobia [7–35]		8.93	3.12	7–27	8
Depression [16–80]		26.43	9.82	16–72	20–23
Somatization [12–60]		20.74	7.59	12–59	15–18
Insufficiency of handling and thoughts [9–45]		15.20	6.10	9–43	11–14
Sensitivity [17–72]		23.99	7.39	17–72	22–26
Hostility [6–30]		7.68	2.65	6–26	7–8
Sleep [3–15]		7.428	3.51	3–15	4–5
Other [9–45]		12.05	3.85	9–32	
Psychoneurotic [90–450]		137.91	39.93	90–360	113–123

*De Ridder et al. (22)*.

*Arrindell and Ettema (24)*.

### Correlation Between Coping and Tinnitus Burden

To study the relationship between tinnitus burden and different coping styles we analyzed the correlation between the THI, TQ, and CISS sub scores (Table 2.2). A statistical significant positive correlation for both sexes was found with “emotional-orientated coping” and both tinnitus questionnaires. A significant negative correlation for both sexes was found with “task-orientated coping.” “Avoidance-orientated coping” had a positive significant correlation in men using THI and TQ. In females when using the TQ no statistical significance was found (B = −0.03. 95% CI = −0.13–0.07, *p* = 0.529). “Avoidance-orientated coping” is divided in two subgroups: “distraction” and “social diversion” “Distraction” demonstrates a significant positive correlation for both sexes. “social diversion” on the other hand was only statistical significantly (negatively) correlated in females.

**Table 2.2:** Results.

		**THI**			**TQ**		
**CISS (*N* = 306)**	**sex**	**B**	**95% Cl**	***P***	**B**	**95% CI**	***P***
Task-orientated coping	M	−0.28	−0.37– −0.20	<0.01	−0.24	−0.30– −0.18	<0.01
	F	−0.26	−0.38– −0.15	<0.01	−0.25	−0.34– −0.17	<0.01
Emotional–orientated coping	M	0.61	0.52–0.70	<0.01	0.23	0.16–0.30	<0.01
	F	0.77	0.64–0.89	<0.01	0.38	0.29–0.48	<0.01
Avoidance-orientated coping	M	0.29	0.19–0.39	<0.01	0.21	0.14–0.28	<0.01
	F	0.17	0.04–0.31	0.01	−0.03	−0.13–0.07	0.53
Distraction	M	0.64	0.46–0.82	<0.01	0.40	0.27–0.53	<0.01
	F	0.76	0.55–0.97	<0.01	0.38	0.22– −0.54	<0.01
Social diversion	M	0.04	−0.22–0.30	0.75	−0.14	−0.33–0.04	0.13
	F	−0.75	−1.06– −0.44	<0.01	−0.96	−1.19– −0.74	<0.01

*Outcome of linear regression analysis of CISS scores and tinnitus questionnaires (THI, TQ). B, Regression coefficient; 95% Ci, 95% confidence interval; F, female; M, male. Missing data was imputed. A total of 306 patients were included in the analysis (207 males, 99 females)*.

### Correlation Between Psychopathological Factors and Tinnitus Burden

To study the relationship between psychopathological factors and tinnitus handicap we analyzed the correlation between THI, TQ, and SCL-90R scores. Statistically significant, positive correlations were found between tinnitus handicap and “anxiety,” “agoraphobia,” “depression,” “insufficiency of handling,” “distrust & personal sensitivity,” “hostility,” and “sleep problems” for both the THI and TQ outcomes (Table 2.3).

**Table 2.3:** Results.

	**THI**			**TQ**		
**SCL-90-R (*N* = 308)**	**B**	**95% Cl**	***P***	**B**	**95% CI**	***P***
Anxiety	2.07	1.97–2.18	<0.01	1.27	1.19–1.35	<0.01
Agoraphobia	3.14	2.92–3.37	<0.01	1.98	1.81–2.15	<0.01
Depression	1.56	1.50–1.62	<0.01	0.97	0.92–1.02	<0.01
Somatization	1.33	1.23–1.42	<0.01	0.97	0.90–1.03	<0.01
Insufficiency of handling	1.82	1.71–1.93	<0.01	1.06	0.97–1.15	<0.01
Distrust & personal sensitivity	1.32	1.22–1.41	<0.01	0.76	0.69–0.83	<0.01
Hostility	3.39	3.11–3.66	<0.01	1.96	1.76–2.17	<0.01
Sleep problems	3.20	3.00–3.39	<0.01	2.44	2.30–2.58	<0.01

*Outcome of linear regression analysis on SCL-90-R subscales and Tinnitus questionnaires: Tinnitus Handicap Inventory (THI) and Tinnitus Questionnaire (TQ). B, Regression coefficient; 95% CI, 95% confidence interval. Missing data imputed*.

## Discussion

In this cross-sectional study we investigated the correlation between perceived tinnitus burden, used coping strategies and psychopathological characteristics in chronic tinnitus patients visiting a tertiary tinnitus clinic.

The results indicate that patients who experience a higher tinnitus burden more frequently use emotional-based and distraction-based coping strategies as measured by the CISS questionnaire than patients with a lower tinnitus burden. In contrast, task-orientated coping was less frequently used in patients with a higher tinnitus burden. These results should be interpreted with caution, because the effect sizes were small. These numbers indicate that, for example, a 10 point increase on a specific coping subscale is related to about eight point increase on the tinnitus burden scale. Therefore, while statistical significant, the importance of these findings for the tinnitus patient can be discussed. Possible other factors are more important. In previous studies similar results were seen with the demonstration of more passive or avoidance-based strategies in relation to a higher tinnitus burden (14–16, 18, 26).

People develop their coping style during their life before and/or after the onset of tinnitus. The relation between tinnitus burden and specific coping styles can be explained by the reaction these coping strategies cause. Until the day a cure for tinnitus is found, it remains a chronic condition to which patients need to learn to habituate and accommodate. Coping strategies that allow patients to recognize and experience the symptoms cause more acceptance, in contrast to passive and avoiding coping strategies which result in fight or flight reactions (6, 18). On the short term, the latter strategies seem effective, but due to the chronic nature of tinnitus they cause a continuing stress reaction instead of acceptance and adaptation. Passive, controlling and avoiding coping strategies could have a major influence on habituation failure, which can result in chronic tinnitus with a high experienced tinnitus burden (6, 27).

When investigating psychopathological characteristics and tinnitus burden, statistical significant positive correlations were found between tinnitus handicap and depressive symptoms which is in line with previous studies (28). However, in our study severe depressive symptoms were less prevalent (17.3% scored “very high” by the SCL-90R depression sub score) compared to reports out of similar settings; a prevalence of up to 60% of patients with a major depressive disorder were reported by usage of a formal psychiatric diagnostic interview (29). This difference could be explained by the fact that SCL-90R is originally designed to screen for psychological pathologies rather than diagnose them (29–31).

The relation we found between tinnitus handicap and coping could (partly) explain why cognitive behavioral therapy can positively influence quality of life in tinnitus patients (32). When coping strategies are influenced by the applied cognitive behavioral therapy, more favorable coping can result in decreased tinnitus related problems and higher quality of life. Though, it must be kept in mind that beside coping strategies the experienced tinnitus distress is influenced by many other psychological aspects in life, complicating statements about a direct relation between coping and tinnitus handicap (6, 33).

### Strengths and Limitations in Study Design

In this study we used a validated and internationally used questionnaire to assess coping strategies in relation to tinnitus burden. Therefore, a large cohort of chronic tinnitus patients was used with a prospective study design. › data was imputed to minimalize the influence of missing data (34). However, there are several limitations. First of all, we used self-rating questionnaires to assess coping and psychopathological symptoms instead of standardized interviews. The latter is more objective to diagnose mental disorders according to the definitions and criteria of ICD-10 and DSM-V. Thereby, interpretation of outcomes out of this study should be done cautiously because of the screening nature of the questionnaires. Also we did use the THI and TQ to measure tinnitus related effects instead of using the Tinnitus Functional Index (TFI) (35), which is considered as the most recent standard. This is due to the fact that this study was designed before the TFI was developed. Most importantly, because of the cross-sectional design of this study it is not possible to follow patients over a period of time to evaluate if (treatment related) changes in coping style or psychological dimensions results in a change in the experienced tinnitus burden.

## Conclusion

In this study population of chronic tinnitus patients visiting a tinnitus care group of a tertiary center, a significant positive correlation between tinnitus handicap and emotional-orientated coping and distraction-oriented coping was found. Also, a negative correlation with task-orientated coping was identified. A higher tinnitus burden also had a significant positive correlation with anxiety, agoraphobia, depression, somatization, insufficiency of handling and thoughts, sensitivity, hostility, and sleeping problems. The found effect sizes were small. This insight in coping strategies in a group of patients with high tinnitus burden is useful for counseling patients. As tinnitus coping strategy might be related to the extent and success of habituation, this outcome could be of interest to optimize tinnitus treatments in the near future.

## Data Availability Statement

The raw data supporting the conclusions of this article will be made available by the authors, without undue reservation.

## Ethics Statement

The studies involving human participants were reviewed and approved by UMCU ethics commission. The patients/participants provided their written informed consent to participate in this study.

## Author Contributions

SM and IS analyzed the data. SM, IS, AL, and AS drafted the manuscript. All authors revised the manuscript, contributed to the interpretation of the results, approved the final version of this study, and contributed to the data acquisition.

## Conflict of Interest

The authors declare that the research was conducted in the absence of any commercial or financial relationships that could be construed as a potential conflict of interest.
